# Supplementary material for: Circulating miR-499a and miR-125b as Potential Predictors of Left Ventricular Ejection Fraction Improvement after Cardiac Resynchronization Therapy
Source: Cells. 2022 Jan 13;11(2):271. doi: 10.3390/cells11020271 (PMC8773679; doi:10.3390/cells11020271)
Supplement: Supplementary file 1 [file cells-11-00271-s001.zip › cells-1489898-supplementary.pdf]

Supplementary Table S1: Basal miRNAs changes in responders vs non-responders.

	P value	Mean of Non-responders	Mean of Responders	Difference	SE of difference	Quality
hsa-let-7a-5p	0.885	4.827	5.204	-0.376	2.572	OK
hsa-let-7b-5p	0.706	15.446	18.949	-3.503	9.183	OK
hsa-let-7c-5p	0.280	1.257	3.322	-2.065	1.865	OK
hsa-let-7d-5p	0.512	4.178	6.181	-2.003	3.009	OK
hsa-let-7e-5p	0.592	2.628	1.884	0.744	1.369	OK
hsa-let-7f-5p	0.442	1.133	0.643	0.490	0.626	OK
hsa-miR-1-3p	0.647	0.145	0.125	0.020	0.043	OK
hsa-miR-100-5p	0.596	0.513	0.626	-0.113	0.210	OK
hsa-miR-103a-3p	0.757	0.234	0.193	0.041	0.131	OK
hsa-miR-107	0.037*	0.130	0.061	0.069	0.031	OK
hsa-miR-10b-5p	0.529	0.113	0.094	0.019	0.030	OK
hsa-miR-122-5p	0.966	7.027	6.736	0.291	6.725	OK
hsa-miR-124-3p	0.082	0.170	0.087	0.083	0.046	OK
hsa-miR-125a-5p	0.367	0.974	1.338	-0.365	0.396	OK
hsa-miR-125b-5p	0.141	0.382	0.794	-0.413	0.271	OK
hsa-miR-126-3p	0.106	3.815	17.158	-13.343	7.924	OK
hsa-miR-130a-3p	0.099	0.268	0.567	-0.298	0.174	OK
hsa-miR-133a-3p	0.033	0.095	0.044	0.051	0.022	A
hsa-miR-133b	0.038	0.095	0.046	0.049	0.022	A
hsa-miR-140-5p	0.208	0.102	34.336	-34.234	26.401	OK

hsa-miR-142-3p	0.214	0.177	52.452	-52.275	40.904	OK
hsa-miR-143-3p	0.265	0.101	0.294	-0.193	0.169	OK
hsa-miR-144-3p	0.271	0.213	0.346	-0.133	0.118	OK
hsa-miR-145-5p	0.729	0.244	0.219	0.025	0.072	OK
hsa-miR-146a-5p	0.287	0.667	1.126	-0.459	0.421	OK
hsa-miR-149-5p	0.307	0.095	0.313	-0.218	0.208	A
hsa-miR-150-5p	0.288	4.722	8.002	-3.280	3.016	OK
hsa-miR-155-5p	0.242	0.103	0.728	-0.625	0.521	OK
hsa-miR-15b-5p	0.884	6.188	5.685	0.503	3.423	OK
hsa-miR-16-5p	0.481	23.497	35.660	-12.163	16.962	OK
hsa-miR-106a-5p hsa-miR-17-5p	0.117	0.454	0.961	-0.507	0.311	OK
hsa-miR-181a-5p	0.319	0.102	0.278	-0.176	0.172	OK
hsa-miR-181b-5p	0.183	0.193	0.702	-0.508	0.370	OK
hsa-miR-182-5p	0.086	0.206	0.057	0.148	0.083	OK
hsa-miR-183-5p	0.365	0.469	0.158	0.311	0.337	OK
hsa-miR-185-5p	0.989	0.800	0.807	-0.007	0.522	OK
hsa-miR-18b-5p	0.692	0.110	0.136	-0.026	0.064	OK
hsa-miR-195-5p	0.711	18.656	23.161	-4.505	12.029	OK
hsa-miR-199a-5p	0.331	0.097	0.224	-0.127	0.128	OK
hsa-miR-206	0.244	0.103	0.562	-0.459	0.384	OK
hsa-miR-208a-3p	0.248	0.095	0.744	-0.649	0.548	A
hsa-miR-208b-3p	0.226	0.095	1.898	-1.803	1.451	A
hsa-miR-21-5p	0.904	27.804	29.057	-1.253	10.309	OK
hsa-miR-210-3p	0.271	0.097	0.468	-0.372	0.330	OK
hsa-miR-214-3p	0.286	0.096	0.349	-0.253	0.232	OK

hsa-miR-22-3p	0.139	0.469	3.510	-3.041	1.984	OK
hsa-miR-221-3p	0.233	0.411	0.732	-0.321	0.262	OK
hsa-miR-222-3p	0.195	0.528	8.470	-7.942	5.948	OK
hsa-miR-223-3p	0.473	19.092	26.302	-7.210	9.884	OK
hsa-miR-224-5p	0.266	0.099	0.248	-0.149	0.131	OK
hsa-miR-23a-3p	0.402	6.975	9.253	-2.278	2.666	OK
hsa-miR-23b-3p	0.422	0.880	1.148	-0.268	0.327	OK
hsa-miR-24-3p	0.193	0.650	1.111	-0.461	0.344	OK
hsa-miR-25-3p	0.194	13.531	26.764	-13.233	9.901	OK
hsa-miR-26a-5p	0.940	5.019	4.807	0.211	2.758	OK
hsa-miR-26b-5p	0.612	3.341	2.318	1.024	1.993	OK
hsa-miR-27a-3p	0.232	1.561	2.799	-1.238	1.008	OK
hsa-miR-27b-3p	0.533	0.816	1.116	-0.300	0.475	OK
hsa-miR-29a-3p	0.206	0.311	7.431	-7.120	5.467	OK
hsa-miR-29b-3p	0.517	0.445	0.276	0.169	0.257	OK
hsa-miR-29c-3p	0.316	0.616	0.944	-0.328	0.320	OK
hsa-miR-302a-3p	0.222	0.095	0.064	0.031	0.025	A
hsa-miR-302b-3p	0.042*	0.095	0.048	0.047	0.022	A
hsa-miR-30a-5p	0.229	1.127	1.835	-0.708	0.572	OK
hsa-miR-30c-5p	0.379	0.469	0.673	-0.205	0.228	OK
hsa-miR-30d-5p	0.086	0.765	1.407	-0.642	0.358	OK
hsa-miR-30e-5p	0.187	0.960	13.015	-12.055	8.864	OK
hsa-miR-31-5p	0.220	0.095	1.602	-1.507	1.196	A
hsa-miR-320a	0.926	4.116	4.287	-0.171	1.832	OK
hsa-miR-328-3p	0.425	1.047	2.092	-1.045	1.286	OK

hsa-miR-342-3p	0.443	0.965	1.273	-0.309	0.395	OK
hsa-miR-365a-3p hsa-miR-365b-3p	0.230	0.197	0.453	-0.256	0.208	OK
hsa-miR-378a-3p	0.833	0.120	0.116	0.005	0.022	OK
hsa-miR-423-3p	0.239	0.113	0.158	-0.045	0.037	OK
hsa-miR-424-5p	0.143	0.665	1.166	-0.500	0.330	OK
hsa-miR-451a	0.200	70.014	141.105	-71.091	53.863	OK
hsa-miR-486-5p	0.428	179.933	240.608	-60.675	75.210	OK
hsa-miR-494-3p	0.051	0.096	0.050	0.046	0.022	OK
hsa-miR-499a-5p	0.033*	0.097	0.048	0.049	0.022	OK
hsa-miR-7-5p	0.739	0.549	0.438	0.111	0.328	OK
hsa-miR-92a-3p	0.117	32.346	60.851	-28.505	17.487	OK
hsa-miR-93-5p	0.272	0.727	1.232	-0.505	0.449	OK
hsa-miR-98-5p	0.256	0.116	0.339	-0.223	0.192	OK
hsa-miR-99a-5p	0.681	0.264	0.303	-0.039	0.095	OK

Supplementary Table S2: 6 months miRNAs changes in responders vs non-responders.

	P value	Mean of Non-responders	Mean of Responders	Difference	SE of difference	Quality
hsa-let-7a-5p	0.524	6.505	10.175	-3.670	5.663	OK
hsa-let-7b-5p	0.747	22.259	19.224	3.035	9.279	OK
hsa-let-7c-5p	0.380	1.388	3.484	-2.097	2.338	OK
hsa-let-7d-5p	0.556	5.409	7.598	-2.189	3.663	OK

hsa-let-7e-5p	0.372	2.816	1.995	0.821	0.901	OK
hsa-let-7f-5p	0.967	1.062	1.085	-0.023	0.556	OK
hsa-miR-1-3p	0.286	0.051	0.076	-0.024	0.022	OK
hsa-miR-100-5p	0.902	0.386	0.398	-0.012	0.093	OK
hsa-miR-103a-3p	0.589	0.146	0.178	-0.032	0.058	OK
hsa-miR-107	0.362	0.058	0.047	0.012	0.013	OK
hsa-miR-10b-5p	0.277	0.079	0.062	0.017	0.015	OK
hsa-miR-122-5p	0.779	3.521	3.097	0.424	1.489	OK
hsa-miR-124-3p	0.894	0.078	0.074	0.005	0.034	OK
hsa-miR-125a-5p	0.668	1.186	1.384	-0.198	0.454	OK
hsa-miR-125b-5p	0.580	0.221	0.273	-0.052	0.093	OK
hsa-miR-126-3p	0.346	6.108	15.748	-9.640	10.002	OK
hsa-miR-130a-3p	0.440	0.425	0.301	0.124	0.158	OK
hsa-miR-133a-3p	0.757	0.042	0.038	0.004	0.013	B
hsa-miR-133b	0.757	0.042	0.038	0.004	0.013	B
hsa-miR-140-5p	0.386	0.053	45.586	-45.533	51.454	OK
hsa-miR-142-3p	0.426	0.165	6.899	-6.734	8.287	OK
hsa-miR-143-3p	0.414	0.051	0.179	-0.128	0.153	OK
hsa-miR-144-3p	0.746	0.186	0.166	0.020	0.062	OK
hsa-miR-145-5p	0.403	0.126	0.147	-0.021	0.025	OK
hsa-miR-146a-5p	0.307	1.271	2.038	-0.766	0.732	OK
hsa-miR-149-5p	0.427	0.042	0.166	-0.124	0.153	A
hsa-miR-150-5p	0.997	9.981	9.992	-0.011	3.412	OK
hsa-miR-155-5p	0.392	0.056	0.834	-0.778	0.891	OK
hsa-miR-15b-5p	0.566	9.181	7.249	1.932	3.309	OK

hsa-miR-16-5p	0.446	49.115	35.962	13.153	16.919	OK
hsa-miR-106a-5p hsa-miR-17-5p	0.935	0.650	0.671	-0.021	0.259	OK
hsa-miR-181a-5p	0.487	0.046	0.074	-0.028	0.039	OK
hsa-miR-181b-5p	0.563	0.214	0.348	-0.134	0.229	OK
hsa-miR-182-5p	0.240	0.066	0.049	0.016	0.013	OK
hsa-miR-183-5p	0.072	0.204	0.087	0.117	0.062	OK
hsa-miR-185-5p	0.398	1.055	0.665	0.390	0.452	OK
hsa-miR-18b-5p	0.670	0.060	0.066	-0.006	0.015	OK
hsa-miR-195-5p	0.462	28.371	21.246	7.125	9.505	OK
hsa-miR-199a-5p	0.393	0.042	0.125	-0.083	0.095	A
hsa-miR-206	0.426	0.042	0.372	-0.330	0.406	OK
hsa-miR-208a-3p	0.388	0.042	0.124	-0.082	0.093	A
hsa-miR-208b-3p	0.367	0.042	0.315	-0.273	0.296	A
hsa-miR-21-5p	0.718	28.913	25.854	3.059	8.355	OK
hsa-miR-210-3p	0.454	0.043	0.113	-0.070	0.092	OK
hsa-miR-214-3p	0.438	0.042	0.142	-0.100	0.126	OK
hsa-miR-22-3p	0.568	0.870	1.374	-0.504	0.869	OK
hsa-miR-221-3p	0.663	0.798	0.950	-0.152	0.345	OK
hsa-miR-222-3p	0.418	1.183	2.249	-1.066	1.291	OK
hsa-miR-223-3p	0.575	46.015	56.946	-10.931	19.202	OK
hsa-miR-224-5p	0.116	0.096	0.236	-0.139	0.085	OK
hsa-miR-23a-3p	0.397	11.066	15.013	-3.947	4.564	OK
hsa-miR-23b-3p	0.528	1.340	1.686	-0.346	0.538	OK
hsa-miR-24-3p	0.348	1.113	1.646	-0.533	0.555	OK
hsa-miR-25-3p	0.284	37.101	20.753	16.348	14.856	OK

hsa-miR-26a-5p	0.324	6.703	10.313	-3.610	3.577	OK
hsa-miR-26b-5p	0.677	3.627	4.372	-0.745	1.761	OK
hsa-miR-27a-3p	0.834	1.898	2.009	-0.111	0.524	OK
hsa-miR-27b-3p	0.677	0.793	0.896	-0.104	0.245	OK
hsa-miR-29a-3p	0.421	0.357	1.403	-1.046	1.276	OK
hsa-miR-29b-3p	0.821	0.391	0.364	0.027	0.118	OK
hsa-miR-29c-3p	0.674	0.670	0.750	-0.080	0.187	OK
hsa-miR-302a-3p	0.789	0.042	0.038	0.003	0.013	A
hsa-miR-302b-3p	0.757	0.042	0.038	0.004	0.013	B
hsa-miR-30a-5p	0.872	1.814	1.920	-0.106	0.654	OK
hsa-miR-30c-5p	0.653	0.928	1.093	-0.165	0.362	OK
hsa-miR-30d-5p	0.824	1.284	1.391	-0.107	0.477	OK
hsa-miR-30e-5p	0.412	1.394	3.123	-1.728	2.063	OK
hsa-miR-31-5p	0.380	0.042	0.286	-0.243	0.272	OK
hsa-miR-320a	0.275	6.955	3.157	3.798	3.389	OK
hsa-miR-328-3p	0.435	0.475	0.850	-0.375	0.470	OK
hsa-miR-342-3p	0.690	1.595	1.769	-0.173	0.428	OK
hsa-miR-365a-3p<br>hsa-miR-365b-3p	0.541	0.122	0.166	-0.043	0.070	OK
hsa-miR-378a-3p	0.407	0.078	0.064	0.013	0.016	OK
hsa-miR-423-3p	0.223	0.122	0.188	-0.066	0.052	OK
hsa-miR-424-5p	0.622	1.008	0.851	0.158	0.315	OK
hsa-miR-451a	0.470	172.594	123.802	48.792	66.325	OK
hsa-miR-486-5p	0.108	309.785	127.445	182.341	108.739	OK
hsa-miR-494-3p	0.661	0.048	0.054	-0.006	0.013	OK
hsa-miR-499a-5p	0.757	0.042	0.038	0.004	0.013	B

hsa-miR-7-5p	0.373	0.553	0.366	0.187	0.205	OK
hsa-miR-92a-3p	0.210	72.371	40.223	32.148	24.854	OK
hsa-miR-93-5p	0.381	1.503	1.022	0.481	0.538	OK
hsa-miR-98-5p	0.188	0.095	0.204	-0.109	0.080	OK
hsa-miR-99a-5p	0.987	0.191	0.190	0.001	0.043	OK

Supplementary Table S3: Longitudinal miRNAs changes in responders group

	P value	Mean of 6 Months	Mean of Basal	Difference	SE of difference	Quality
hsa-let-7a-5p	0.277	10.175	5.204	4.971	4.481	OK
hsa-let-7b-5p	0.972	19.224	18.949	0.276	7.862	OK
hsa-let-7c-5p	0.947	3.484	3.322	0.162	2.418	OK
hsa-let-7d-5p	0.647	7.598	6.181	1.417	3.057	OK
hsa-let-7e-5p	0.890	1.995	1.884	0.112	0.798	OK
hsa-let-7f-5p	0.338	1.085	0.643	0.442	0.453	OK
hsa-miR-1-3p	0.152	0.076	0.125	-0.049	0.033	OK
hsa-miR-100-5p	0.184	0.398	0.626	-0.228	0.167	OK
hsa-miR-103a-3p	0.853	0.178	0.193	-0.015	0.080	OK
hsa-miR-107	0.327	0.047	0.061	-0.014	0.014	OK
hsa-miR-10b-5p	0.172	0.062	0.094	-0.032	0.023	OK
hsa-miR-122-5p	0.412	3.097	6.736	-3.639	4.367	OK
hsa-miR-124-3p	0.674	0.074	0.087	-0.013	0.031	OK
hsa-miR-125a-5p	0.919	1.384	1.338	0.045	0.441	OK

hsa-miR-125b-5p	0.031*	0.273	0.794	-0.521	0.229	OK
hsa-miR-126-3p	0.895	15.748	17.158	-1.410	10.540	OK
hsa-miR-130a-3p	0.042*	0.301	0.567	-0.266	0.124	OK
hsa-miR-133a-3p	0.650	0.038	0.044	-0.006	0.014	B
hsa-miR-133b	0.540	0.038	0.046	-0.008	0.014	A
hsa-miR-140-5p	0.813	45.586	34.336	11.250	47.075	OK
hsa-miR-142-3p	0.225	6.899	52.452	-45.553	36.686	OK
hsa-miR-143-3p	0.555	0.179	0.294	-0.115	0.192	OK
hsa-miR-144-3p	0.051	0.166	0.346	-0.180	0.088	OK
hsa-miR-145-5p	0.142	0.147	0.219	-0.072	0.047	OK
hsa-miR-146a-5p	0.164	2.038	1.126	0.912	0.637	OK
hsa-miR-149-5p	0.509	0.166	0.313	-0.147	0.220	OK
hsa-miR-150-5p	0.562	9.992	8.002	1.990	3.387	OK
hsa-miR-155-5p	0.902	0.834	0.728	0.106	0.844	OK
hsa-miR-15b-5p	0.424	7.249	5.685	1.564	1.925	OK
hsa-miR-16-5p	0.981	35.962	35.660	0.302	12.513	OK
hsa-miR-106a-5p hsa-miR-17-5p	0.271	0.671	0.961	-0.289	0.257	OK
hsa-miR-181a-5p	0.197	0.074	0.278	-0.205	0.154	OK
hsa-miR-181b-5p	0.350	0.348	0.702	-0.354	0.372	OK
hsa-miR-182-5p	0.557	0.049	0.057	-0.008	0.013	OK
hsa-miR-183-5p	0.472	0.087	0.158	-0.071	0.098	OK
hsa-miR-185-5p	0.546	0.665	0.807	-0.142	0.232	OK
hsa-miR-18b-5p	0.221	0.066	0.136	-0.070	0.056	OK
hsa-miR-195-5p	0.815	21.246	23.161	-1.915	8.088	OK
hsa-miR-199a-5p	0.468	0.125	0.224	-0.099	0.135	OK

hsa-miR-206	0.688	0.372	0.562	-0.190	0.468	OK
hsa-miR-208a-3p	0.216	0.124	0.744	-0.620	0.489	OK
hsa-miR-208b-3p	0.235	0.315	1.898	-1.583	1.302	OK
hsa-miR-21-5p	0.612	25.854	29.057	-3.203	6.238	OK
hsa-miR-210-3p	0.246	0.113	0.468	-0.356	0.299	OK
hsa-miR-214-3p	0.372	0.142	0.349	-0.207	0.227	OK
hsa-miR-22-3p	0.264	1.374	3.510	-2.136	1.870	OK
hsa-miR-221-3p	0.511	0.950	0.732	0.217	0.327	OK
hsa-miR-222-3p	0.255	2.249	8.470	-6.221	5.343	OK
hsa-miR-223-3p	0.062	56.946	26.302	30.644	15.706	OK
hsa-miR-224-5p	0.928	0.236	0.248	-0.012	0.132	OK
hsa-miR-23a-3p	0.161	15.013	9.253	5.760	3.989	OK
hsa-miR-23b-3p	0.263	1.686	1.148	0.538	0.470	OK
hsa-miR-24-3p	0.295	1.646	1.111	0.535	0.501	OK
hsa-miR-25-3p	0.496	20.753	26.764	-6.011	8.704	OK
hsa-miR-26a-5p	0.091	10.313	4.807	5.506	3.138	OK
hsa-miR-26b-5p	0.191	4.372	2.318	2.054	1.532	OK
hsa-miR-27a-3p	0.387	2.009	2.799	-0.790	0.898	OK
hsa-miR-27b-3p	0.566	0.896	1.116	-0.220	0.379	OK
hsa-miR-29a-3p	0.232	1.403	7.431	-6.028	4.929	OK
hsa-miR-29b-3p	0.385	0.364	0.276	0.088	0.100	OK
hsa-miR-29c-3p	0.379	0.750	0.944	-0.194	0.217	OK
hsa-miR-302a-3p	0.148	0.038	0.064	-0.025	0.017	OK
hsa-miR-302b-3p	0.480	0.038	0.048	-0.010	0.014	A
hsa-miR-30a-5p	0.887	1.920	1.835	0.086	0.596	OK

hsa-miR-30c-5p	0.202	1.093	0.673	0.419	0.320	OK
hsa-miR-30d-5p	0.968	1.391	1.407	-0.016	0.396	OK
hsa-miR-30e-5p	0.226	3.123	13.015	-9.892	7.985	OK
hsa-miR-31-5p	0.232	0.286	1.602	-1.316	1.077	OK
hsa-miR-320a	0.324	3.157	4.287	-1.130	1.125	OK
hsa-miR-328-3p	0.294	0.850	2.092	-1.242	1.159	OK
hsa-miR-342-3p	0.261	1.769	1.273	0.495	0.431	OK
hsa-miR-365a-3p hsa-miR-365b-3p	0.123	0.166	0.453	-0.287	0.180	OK
hsa-miR-378a-3p	0.002*	0.064	0.116	-0.051	0.015	OK
hsa-miR-423-3p	0.545	0.188	0.158	0.031	0.050	OK
hsa-miR-424-5p	0.288	0.851	1.166	-0.315	0.291	OK
hsa-miR-451a	0.724	123.802	141.105	-17.303	48.495	OK
hsa-miR-486-5p	0.085	127.445	240.608	-113.163	63.155	OK
hsa-miR-494-3p	0.769	0.054	0.050	0.004	0.014	OK
hsa-miR-499a-5p	0.469	0.038	0.048	-0.010	0.013	A
hsa-miR-7-5p	0.629	0.366	0.438	-0.072	0.148	OK
hsa-miR-92a-3p	0.196	40.223	60.851	-20.628	15.559	OK
hsa-miR-93-5p	0.499	1.022	1.232	-0.209	0.305	OK
hsa-miR-98-5p	0.459	0.204	0.339	-0.135	0.180	OK
hsa-miR-99a-5p	0.159	0.190	0.303	-0.113	0.078	OK

Supplementary Table S4: Longitudinal miRNAs changes in non-responders group

	P value	Mean of 6 Months	Mean of Basal	Difference	SE of difference	Quality
hsa-let-7a-5p	0.835	6.505	5.647	0.858	3.975	OK
hsa-let-7b-5p	0.716	22.259	17.204	5.055	13.423	OK
hsa-let-7c-5p	0.976	1.388	1.418	-0.030	0.970	OK
hsa-let-7d-5p	0.933	5.409	5.039	0.370	4.246	OK
hsa-let-7e-5p	0.915	2.816	3.031	-0.214	1.947	OK
hsa-let-7f-5p	0.763	1.062	1.339	-0.277	0.887	OK
hsa-miR-1-3p	0.095	0.051	0.132	-0.080	0.043	OK
hsa-miR-100-5p	0.222	0.386	0.582	-0.196	0.148	OK
hsa-miR-103a-3p	0.574	0.146	0.240	-0.094	0.161	OK
hsa-miR-107	0.173	0.058	0.114	-0.055	0.037	OK
hsa-miR-10b-5p	0.526	0.079	0.093	-0.014	0.021	OK
hsa-miR-122-5p	0.434	3.521	8.453	-4.932	5.994	OK
hsa-miR-124-3p	0.294	0.078	0.140	-0.062	0.055	OK
hsa-miR-125a-5p	0.866	1.186	1.118	0.068	0.389	OK
hsa-miR-125b-5p	0.237	0.221	0.421	-0.200	0.157	OK
hsa-miR-126-3p	0.369	6.108	4.599	1.510	1.586	OK
hsa-miR-130a-3p	0.608	0.425	0.283	0.143	0.268	OK
hsa-miR-133a-3p	0.179	0.042	0.071	-0.029	0.020	A
hsa-miR-133b	0.179	0.042	0.071	-0.029	0.020	A
hsa-miR-140-5p	0.059	0.053	0.079	-0.026	0.012	OK
hsa-miR-142-3p	0.888	0.165	0.171	-0.006	0.042	OK

hsa-miR-143-3p	0.165	0.051	0.078	-0.026	0.017	OK
hsa-miR-144-3p	0.822	0.186	0.215	-0.029	0.125	OK
hsa-miR-145-5p	0.132	0.126	0.253	-0.127	0.076	OK
hsa-miR-146a-5p	0.214	1.271	0.770	0.502	0.372	OK
hsa-miR-149-5p	0.179	0.042	0.071	-0.029	0.020	A
hsa-miR-150-5p	0.083	9.981	5.537	4.444	2.246	OK
hsa-miR-155-5p	0.194	0.056	0.081	-0.025	0.017	OK
hsa-miR-15b-5p	0.787	9.181	7.451	1.731	6.195	OK
hsa-miR-16-5p	0.475	49.115	28.539	20.577	27.469	OK
hsa-miR-106a-5p<br>hsa-miR-17-5p	0.729	0.650	0.508	0.142	0.397	OK
hsa-miR-181a-5p	0.054	0.046	0.080	-0.034	0.015	OK
hsa-miR-181b-5p	0.734	0.214	0.191	0.023	0.064	OK
hsa-miR-182-5p	0.262	0.066	0.206	-0.140	0.116	OK
hsa-miR-183-5p	0.489	0.204	0.528	-0.325	0.447	OK
hsa-miR-185-5p	0.898	1.055	0.932	0.123	0.929	OK
hsa-miR-18b-5p	0.121	0.060	0.089	-0.029	0.017	OK
hsa-miR-195-5p	0.763	28.371	22.703	5.668	18.183	OK
hsa-miR-199a-5p	0.133	0.042	0.073	-0.031	0.019	A
hsa-miR-206	0.095	0.042	0.080	-0.038	0.020	OK
hsa-miR-208a-3p	0.179	0.042	0.071	-0.029	0.020	A
hsa-miR-208b-3p	0.179	0.042	0.071	-0.029	0.020	A
hsa-miR-21-5p	0.795	28.913	33.277	-4.364	16.257	OK
hsa-miR-210-3p	0.142	0.043	0.073	-0.030	0.018	OK
hsa-miR-214-3p	0.162	0.042	0.072	-0.030	0.019	OK
hsa-miR-22-3p	0.365	0.870	0.528	0.342	0.356	OK

hsa-miR-221-3p	0.199	0.798	0.457	0.340	0.243	OK
hsa-miR-222-3p	0.135	1.183	0.601	0.582	0.350	OK
hsa-miR-223-3p	0.111	46.015	22.707	23.308	13.022	OK
hsa-miR-224-5p	0.356	0.096	0.075	0.021	0.022	OK
hsa-miR-23a-3p	0.271	11.066	8.206	2.860	2.418	OK
hsa-miR-23b-3p	0.340	1.340	1.031	0.309	0.305	OK
hsa-miR-24-3p	0.265	1.113	0.749	0.364	0.304	OK
hsa-miR-25-3p	0.328	37.101	16.071	21.030	20.206	OK
hsa-miR-26a-5p	0.845	6.703	6.008	0.695	3.452	OK
hsa-miR-26b-5p	0.891	3.627	4.004	-0.377	2.673	OK
hsa-miR-27a-3p	0.950	1.898	1.863	0.036	0.549	OK
hsa-miR-27b-3p	0.695	0.793	0.952	-0.160	0.393	OK
hsa-miR-29a-3p	0.723	0.357	0.332	0.024	0.067	OK
hsa-miR-29b-3p	0.780	0.391	0.498	-0.107	0.370	OK
hsa-miR-29c-3p	0.924	0.670	0.708	-0.038	0.381	OK
hsa-miR-302a-3p	0.179	0.042	0.071	-0.029	0.020	A
hsa-miR-302b-3p	0.179	0.042	0.071	-0.029	0.020	A
hsa-miR-30a-5p	0.527	1.814	1.332	0.482	0.729	OK
hsa-miR-30c-5p	0.070	0.928	0.527	0.400	0.191	OK
hsa-miR-30d-5p	0.473	1.284	0.890	0.394	0.524	OK
hsa-miR-30e-5p	0.632	1.394	1.127	0.267	0.536	OK
hsa-miR-31-5p	0.181	0.042	0.071	-0.028	0.019	A
hsa-miR-320a	0.694	6.955	4.912	2.044	5.010	OK
hsa-miR-328-3p	0.277	0.475	0.993	-0.518	0.444	OK
hsa-miR-342-3p	0.120	1.595	1.077	0.518	0.297	OK

hsa-miR-365a-3p<br>hsa-miR-365b-3p	0.490	0.122	0.195	-0.073	0.100	OK
hsa-miR-378a-3p	0.226	0.078	0.102	-0.024	0.018	OK
hsa-miR-423-3p	0.137	0.122	0.093	0.030	0.018	OK
hsa-miR-424-5p	0.587	1.008	0.768	0.241	0.425	OK
hsa-miR-451a	0.354	172.594	84.755	87.839	89.221	OK
hsa-miR-486-5p	0.507	309.785	212.000	97.785	140.666	OK
hsa-miR-494-3p	0.235	0.048	0.072	-0.023	0.018	OK
hsa-miR-499a-5p	0.148	0.042	0.073	-0.031	0.019	A
hsa-miR-7-5p	0.885	0.553	0.626	-0.073	0.487	OK
hsa-miR-92a-3p	0.328	72.371	38.815	33.556	32.235	OK
hsa-miR-93-5p	0.467	1.503	0.843	0.660	0.865	OK
hsa-miR-98-5p	0.943	0.095	0.097	-0.002	0.024	OK
hsa-miR-99a-5p	0.236	0.191	0.277	-0.086	0.067	OK

A: This gene's average threshold cycle is relatively high (> 30) in either the control or the test sample, and is reasonably low in the other sample (< 30).
These data mean that the gene's expression is relatively low in one sample and reasonably detected in the other sample suggesting that the actual fold-change value is at least as large as the calculated and reported fold-change result.
This fold-change result may also have greater variations if p value > 0.05; therefore, it is important to have a sufficient number of biological replicates to validate the result for this gene.
B: This gene's average threshold cycle is relatively high (> 30), meaning that its relative expression level is low, in both control and test samples, and the p-value for the fold-change is either unavailable or relatively high (p > 0.05).
This fold-change result may also have greater variations; therefore, it is important to have a sufficient number of biological replicates to validate the result for this gene.

Supplementary Table S5: AUC values of basal altered miRNAs expression in responders vs non-responders.

	Area	Std. Error	95% C.I.	P value
hsa-miR-107	0.740	0.101	0.543 to 0.937	0.043*
hsa-miR-302b-3p	0.727	0.109	0.515 to 0.940	0.055
hsa-miR-499a-5p	0.747	0.106	0.540 to 0.954	0.037*

Supplementary Table S6: AUC values of longitudinal altered miRNAs expression in responders

	Area	Std. Error	95% C.I.	P value
hsa-miR-125b-5p	0.735	0.097	0.546 to 0.924	0.035*
hsa-miR-130a-3p	0.699	0.109	0.486 to 0.912	0.073
hsa-miR-378a-3p	0.816	0.083	0.654 to 0.979	0.004*

Supplementary Table S7: Sensitivity and Specificity of basal altered miRNAs expression in responders vs non-responders.

	Sensitivity %	95% CI	Specificity %	95% CI
hsa-miR-107	63.64	35.38% to 84.83%	78.57	52.41% to 92.43%
hsa-miR-302b-3p	64.29	38.76% to 83.66%	81.82	52.30% to 96.77%
hsa-miR-499a-5p	54.55	28.01% to 78.73%	85.71	60.06% to 97.46%

Supplementary Table S8: Sensitivity and Specificity of longitudinal altered miRNAs expression in responders

	Sensitivity %	95% CI	Specificity %	95% CI
hsa-miR-125b-5p	42.86	21.38% to 67.41%	92.86	68.53% to 99.63%
hsa-miR-130a-3p	100.0	78.47% to 100.0%	57.14	32.59% to 78.62%
hsa-miR-378a-3p	57.14	28.86% to 82.34%	85.71	57.19% to 98.22%

Supplementary Table S9: Basal Real-time RT-PCR cycle threshold (Ct) values of 84 cardio-miRs in responder patients

miRNA ID	Responders Baseline													
	**1**	**2**	**3**	**4**	**5**	**6**	**7**	**8**	**9**	**10**	**11**	**12**	**13**	**14**
hsa-let-7a-5p	19.84191	20.0035	21.78521	18.33104	26.02479	24.92978	24.62279	22.74035	26.38322	22.14805	24.46670	23.45431	19.36229	27.37305
hsa-let-7b-5p	19.88526	19.71372	19.01192	17.89871	25.64895	22.02559	21.97262	20.59451	23.67828	20.22782	22.88947	20.55793	16.79912	25.52193
hsa-let-7c-5p	19.42285	19.34845	23.25462	21.59233	28.54032	27.13652	26.08209	24.78808	28.01401	23.50439	26.30528	25.20018	21.16009	27.13247
hsa-let-7d-5p	19.23731	19.24346	21.22658	19.33075	26.07455	23.8795	23.54721	22.74017	25.58212	22.20951	24.79937	22.42504	19.6998	29.19573
hsa-let-7e-5p	30.01877	30.87293	22.0803	19.52586	27.19405	26.08417	25.75033	23.77848	27.45049	23.03729	25.63599	24.65787	20.31588	27.48568
hsa-let-7f-5p	28.59197	30.27145	23.96519	20.01565	28.40646	27.41692	27.02485	25.28643	29.77111	24.87944	26.80365	26.94757	22.49457	30.02407
hsa-miR-1-3p	26.15889	29.13614	25.96546	23.85659	32.81236	31.96589	30.9774	27.50269	32.68694	25.50664	28.60281	29.34673	28.78388	-
hsa-miR-100-5p	-	36.48434	22.98689	21.09347	27.96393	28.5202	26.95949	24.34313	27.57453	23.67261	25.65108	26.96597	24.95645	30.38845
hsa-miR-103a-3p	27.88747	31.53689	26.01816	21.52744	29.43791	28.2178	28.67173	27.24687	29.62132	28.26304	30.13565	28.16726	25.21449	35.01258
hsa-miR-107	30.19726	30.65397	28.84723	24.41639	32.49769	30.7644	30.98152	29.72545	31.47517	31.53936	32.28994	31.18333	27.01526	-
hsa-miR-10b-5p	-	38.24860	25.50524	24.80085	30.42347	31.37139	30.71275	27.01854	31.05729	26.71885	31.88744	29.93233	28.69667	35.20304
hsa-miR-122-5p	38.46095	38.79723	19.82414	20.24732	27.94337	27.36003	24.27572	22.34441	26.16295	18.39364	22.98630	25.86515	22.66536	29.1467
hsa-miR-124-3p	24.74027	26.09708	28.75962	28.02841	29.57519	29.41669	30.36493	30.54312	31.11117	30.38575	29.47168	29.94108	29.28939	29.86893
hsa-miR-125a-5p	25.28661	26.98355	21.82751	20.35802	27.48086	27.751	26.07017	23.03413	26.31552	22.68647	25.21437	25.23791	23.29496	27.36829
hsa-miR-125b-5p	21.68091	22.39346	23.69135	21.86540	28.71359	29.83724	27.2047	24.82085	28.30634	23.63028	26.40038	27.52872	25.20829	30.44353

hsa-miR-126-3p	16.78427	17.36069	19.5423	17.25346	25.06231	25.1079	23.61317	20.80079	25.11842	21.19662	22.86982	22.83431	20.95471	27.4782
hsa-miR-130a-3p	23.49839	24.31251	24.29385	21.78262	29.40453	27.13161	26.03007	24.75671	29.28528	26.54833	28.53514	25.57723	22.67745	35.27981
hsa-miR-133a-3p	31.45377	31.44252	32.23641	30.95988	36.57259	-	-	36.71846	-	34.46545	34.93331	-	-	-
hsa-miR-133b	27.76724	29.76323	32.98231	30.62265	-	-	-	34.63663	-	35.66105	-	34.53553	-	-
hsa-miR-140-5p	15.02599	15.80897	28.06902	25.20641	-	32.42005	32.58796	28.22155	32.36642	29.60947	31.19728	30.57054	28.65279	-
hsa-miR-142-3p	14.68078	14.99658	24.59681	20.26462	29.05801	29.92316	28.2914	25.88765	29.09242	25.67191	28.20863	28.368	28.9574	33.64798
hsa-miR-143-3p	22.34199	22.96559	27.80805	24.6336	-	33.39125	30.19004	27.62456	34.62107	28.21034	29.68711	29.34372	31.86693	36.1478
hsa-miR-144-3p	24.76432	25.8177	23.90718	21.48969	31.45402	27.1004	27.18943	25.52481	29.30063	26.4857	29.44157	26.42274	26.36722	35.26258
hsa-miR-145-5p	26.13858	27.24134	26.55052	22.32615	27.58484	27.97082	27.63689	26.87873	28.48023	27.28841	28.25653	27.49449	28.17701	31.43414
hsa-miR-146a-5p	24.94755	26.37954	22.7501	19.18675	27.2261	26.57445	27.17826	23.78733	27.2079	24.05903	25.19818	24.97361	24.10233	30.14289
hsa-miR-149-5p	21.70731	23.16448	35.89579	34.28728	-	35.06697	36.10102	-	-	36.60941	-	36.06864	-	-
hsa-miR-150-5p	25.56588	25.18984	18.85218	18.48367	25.41242	24.02359	23.94691	21.05849	23.11858	20.05791	24.91524	23.08326	19.24954	27.04442
hsa-miR-155-5p	20.45612	21.73005	28.3755	25.93137	34.79484	33.69769	30.90395	30.78849	33.15408	28.94461	32.66084	32.91734	28.28863	33.0918
hsa-miR-15b-5p	31.53004	32.45306	21.18054	18.06867	25.05935	23.47934	22.88169	21.88479	25.2736	21.6461	23.37335	21.93324	19.19765	27.96061
hsa-miR-16-5p	21.38821	22.34719	18.2124	16.48274	23.93932	21.50247	20.1279	18.09906	22.49489	19.30497	21.34988	18.42423	16.75351	27.55341
hsa-miR-106a-5p hsa-miR-17-5p	21.44151	22.65983	23.26455	20.78955	28.6114	27.46397	26.00477	24.76833	28.0327	24.90981	27.63202	25.05266	23.38988	31.44881
hsa-miR-181a-5p	22.06775	23.23176	29.40321	25.44801	33.24712	31.66862	34.48433	31.72371	31.53551	30.31976	32.63215	32.23122	28.8564	35.96242
hsa-miR-181b-5p	20.99444	21.99187	24.35049	23.61367	31.53049	28.70047	28.52634	26.53133	28.69124	26.00384	28.60162	27.39987	25.64961	31.42443
hsa-miR-182-5p	33.91987	33.99507	28.70471	25.77531	31.15103	33.3937	31.14518	30.49488	32.85178	29.25071	31.20394	31.98878	26.74165	-
hsa-miR-183-5p	35.9649	-	27.87576	26.94693	32.92858	34.1241	30.9359	28.23702	31.71801	28.32008	32.55724	28.7249	23.00968	32.87954
hsa-miR-185-5p	22.41039	23.20716	23.01648	20.95054	30.04265	27.33813	25.97054	24.98705	28.04057	24.49253	27.96551	24.93134	23.74715	-
hsa-miR-18b-5p	23.4579	24.64703	29.94412	25.83877	30.99129	29.79868	34.26055	30.57401	32.13649	30.41582	30.01932	29.7431	28.5822	35.04238
hsa-miR-195-5p	23.49493	24.80106	18.88162	17.15051	24.74492	22.14992	20.60106	18.68317	23.25022	20.19385	21.91696	19.0956	17.2327	28.46631
hsa-miR-199a-5p	22.4414	23.73414	29.99424	24.32582	31.85142	-	33.85891	29.78487	-	31.90337	-	32.26897	30.98636	35.92517
hsa-miR-206	20.98006	22.02368	27.63244	26.28068	31.91676	30.45434	29.90372	28.71449	33.39438	29.33521	28.98229	30.27145	30.1795	33.17344
hsa-miR-208a-3p	20.32714	21.79168	35.83442	36.06089	-	-	-	-	33.95532	-	-	-	-	-
hsa-miR-208b-3p	18.9491	20.34879	31.40434	32.51118	-	35.52602	34.51469	31.27713	-	32.20971	-	-	-	-

hsa-miR-21-5p	18.14406	18.97987	18.00975	15.60474	23.37229	21.59003	21.71637	19.66512	22.66956	18.6676	20.66750	20.53789	17.96542	25.28289
hsa-miR-210-3p	21.06117	22.49526	31.07907	28.24881	32.06826	31.46582	32.10699	30.83752	32.50336	30.21895	30.82009	33.35324	30.18008	31.49533
hsa-miR-214-3p	21.62971	22.85815	29.42631	28.15084	32.09974	-	32.15071	30.32936	-	29.47375	33.24553	32.72726	33.29314	34.40918
hsa-miR-22-3p	18.36045	20.03364	22.31653	21.16313	28.82558	25.21818	25.5744	23.3336	27.06451	24.7162	26.41494	24.49241	24.56397	36.11221
hsa-miR-221-3p	25.14719	25.83372	23.77501	19.80911	27.97625	26.62608	26.98913	24.53132	28.01094	25.54527	25.82673	25.24319	24.53567	32.88506
hsa-miR-222-3p	16.84674	18.41407	22.4975	20.64586	28.12877	26.1463	26.14159	23.73565	27.03469	24.34161	25.86304	25.09861	23.78974	30.8611
hsa-miR-223-3p	22.02249	23.00664	18.97785	15.04457	22.90857	22.88433	21.65954	19.55541	22.92089	19.16157	21.08635	20.60537	17.12063	24.60914
hsa-miR-224-5p	22.33131	23.78264	28.42504	24.29805	31.11204	30.11805	31.05645	27.93089	30.0905	28.79813	30.38456	28.04324	27.41167	31.66068
hsa-miR-23a-3p	22.13383	23.13215	19.74043	16.65263	24.10624	23.804	22.86187	20.86743	23.94379	20.43876	22.02120	21.93121	20.37003	25.876
hsa-miR-23b-3p	29.27188	30.21341	22.27318	20.20253	26.7926	26.11194	25.79623	23.85543	27.04272	22.98634	25.30584	25.03204	23.48955	29.21096
hsa-miR-24-3p	30.43063	30.88632	22.59534	19.66596	27.06186	27.0001	25.75985	23.69747	27.24778	23.6154	24.96817	24.69759	23.86565	31.29105
hsa-miR-25-3p	17.91409	19.06775	18.69221	18.12583	24.50082	22.11332	20.83778	19.83701	22.46482	19.64668	22.59748	19.89317	16.56706	26.26348
hsa-miR-26a-5p	35.80273	34.92135	20.4379	17.10811	24.87404	24.93596	23.94559	21.98698	25.17189	21.74973	24.08385	23.47675	20.72902	27.76607
hsa-miR-26b-5p	-	35.17791	21.56231	18.17565	26.93649	26.21146	25.64935	23.52712	26.52334	22.82048	25.36018	24.79397	20.79751	28.72496
hsa-miR-27a-3p	27.7078	29.56584	20.65573	18.36308	26.64284	25.95812	24.76007	21.83958	25.82593	21.95552	24.22320	24.18883	23.32488	29.99447
hsa-miR-27b-3p	26.78743	29.95699	22.22773	19.95207	27.64341	27.94409	26.19835	23.76239	27.75556	22.34634	25.41401	25.3832	24.68736	30.90149
hsa-miR-29a-3p	17.62799	17.86415	22.91406	21.54144	29.14391	28.90747	27.1875	24.83515	28.43591	24.39931	27.03975	26.85672	25.93566	31.56988
hsa-miR-29b-3p	28.04684	29.28477	24.54293	22.42576	31.29731	29.79417	27.38745	26.17487	29.48786	25.63511	28.34858	25.98013	24.14673	34.84656
hsa-miR-29c-3p	22.19613	23.82256	22.57417	20.72662	28.73598	28.26199	25.90235	23.89878	27.77225	23.79477	26.67847	25.17364	23.83439	32.6746
hsa-miR-302a-3p	26.18324	26.14338	36.12545	31.9296	-	-	-	-	-	33.62856	32.20512	-	-	-
hsa-miR-302b-3p	28.22818	28.33616	-	33.53281	38.6248	-	-	-	-	36.59692	36.46421	-	35.77745	-
hsa-miR-30a-5p	24.37608	26.04666	21.70911	19.16714	27.51054	26.24777	25.5436	22.69412	26.48641	22.93262	25.12290	24.05621	21.7871	31.48616
hsa-miR-30c-5p	26.93115	29.39772	23.19785	20.11044	27.771	27.1657	26.33229	24.65734	27.77622	24.91205	26.60770	26.32274	23.89086	32.25084
hsa-miR-30d-5p	22.51675	23.85957	22.34905	19.81882	27.64244	26.74527	25.86554	23.27111	26.94595	23.55762	25.92150	24.3739	22.09857	32.70195
hsa-miR-30e-5p	16.75027	17.23229	21.91257	19.60997	27.84048	26.46659	25.80032	23.08204	26.89488	23.19162	25.48366	24.32066	22.40355	31.06473
hsa-miR-31-5p	19.47827	20.27329	32.46037	31.10894	35.56205	32.3735	32.99335	31.94077	33.2868	31.6359	34.30622	34.19646	31.99502	32.18044
hsa-miR-320a	27.81806	28.51754	20.50435	19.24573	26.16527	23.52576	23.71193	22.13495	24.07446	21.64691	24.74474	22.79476	19.86958	29.37034

hsa-miR-328-3p	19.03453	20.96022	25.88325	23.97241	28.09706	26.43894	27.7276	27.25989	28.12236	26.82253	27.15777	28.02489	25.15286	25.06053
hsa-miR-342-3p	-	32.94544	21.89783	20.71504	27.09028	26.10258	25.70812	23.88413	26.41371	22.67694	26.45754	24.73621	22.26271	29.14152
hsa-miR-365a-3p hsa-miR-365b-3p	21.73532	23.34527	24.51941	23.99055	30.50763	31.18228	29.11903	26.23175	30.01678	24.29078	28.41197	29.03189	27.43387	-
hsa-miR-378a-3p	25.59985	26.09543	25.9239	24.61909	32.66703	30.11341	28.98842	27.46117	30.37235	27.44928	31.85860	28.11501	27.99808	-
hsa-miR-423-3p	24.55052	25.09448	26.17591	23.11901	30.74596	28.88722	29.11455	27.66664	29.85272	28.26161	30.18772	28.52233	26.85224	33.9571
hsa-miR-424-5p	26.53323	26.99322	22.01997	21.03025	27.83872	25.94928	24.92384	23.20911	26.34507	23.52522	25.88857	24.35336	23.4445	32.5754
hsa-miR-451a	23.75702	24.72434	15.18877	13.83141	22.30174	19.38697	18.38535	16.16323	20.64807	17.22226	19.72102	16.75515	15.28198	26.1818
hsa-miR-486-5p	27.08547	27.63122	14.79736	15.28224	20.82048	16.63221	17.25022	16.84194	17.88499	16.71562	19.51172	16.49862	14.35624	22.17513
hsa-miR-494-3p	33.98846	39.28199	30.16243	25.25605	31.43753	32.79103	30.9574	34.0424	34.41611	30.00187	30.25745	29.83684	29.85147	36.16228
hsa-miR-499a-5p	35.35032	34.1583	28.81025	27.98655	37.55873	35.00038	34.27072	29.55111	34.11559	29.10333	33.50202	34.0736	32.94487	-
hsa-miR-7-5p	23.65496	24.32796	24.84278	24.24265	29.40058	29.07302	27.95275	25.29252	29.29155	25.27816	28.97482	26.4129	22.4848	30.96707
hsa-miR-92a-3p	18.9827	20.25777	16.65834	16.71164	22.76011	20.11454	19.2554	18.3018	20.05514	18.45877	21.29853	18.46733	15.91632	25.47392
hsa-miR-93-5p	24.00928	24.98227	22.52898	20.69374	27.84538	25.65887	24.99172	23.83352	26.77845	24.22874	26.84008	23.77476	21.94543	34.85024
hsa-miR-98-5p	21.99079	22.93133	27.82218	23.19121	30.92509	31.04847	30.71481	28.41908	32.20228	28.35761	29.66981	30.59597	27.46395	35.0232
hsa-miR-99a-5p	29.02829	29.06496	23.97277	22.16447	28.85898	29.75753	27.73792	25.37887	29.0693	24.84456	26.81895	28.192	26.02437	31.81341
SNORD61	27.63758	28.80558	29.03048	26.59955	34.42818	31.13966	32.78331	30.331	31.68502	29.37387	32.00511	34.18114	30.34297	36.25198
SNORD68	24.32069	25.7618	26.85148	24.28254	31.53528	34.11323	32.674	29.58427	34.61507	27.80394	-	31.26811	27.93411	-
SNORD72	23.34288	25.02406	36.19455	34.44434	37.41567	38.38386	-	-	-	-	-	-	38.15257	-
SNORD95	26.30938	27.5625	26.03077	23.91937	31.1936	30.10253	30.97837	28.28977	29.99331	27.81444	30.53891	29.41245	25.60467	-
SNORD96A	22.37372	24.13906	27.79684	25.94733	-	34.00454	-	30.09497	29.39889	29.29656	32.04503	33.27823	26.803	35.04143
RNU6-6P	21.58214	22.75565	29.57277	26.12281	30.47095	30.59433	-	31.17407	31.86222	30.62344	32.49541	-	30.34327	-
miRTC[a]	20.6421	21.53689	18.75702	18.4029	19.07549	19.10357	19.28181	19.05362	19.86876	19.35296	19.11680	19.51311	21.37097	24.04655
miRTC[a]	22.91082	23.47966	18.7177	18.51817	19.05131	19.03902	19.1266	19.06144	19.77307	19.2812	19.31367	19.6554	21.41609	24.01066
PPC[b]	18.81057	19.22936	19.64822	19.88891	20.00624	19.84029	20.07975	19.66761	19.93348	19.69929	20.12112	20.03439	20.09547	20.60515
PPC[b]	20.48571	21.76894	19.7457	20.14518	19.9891	19.97297	19.82986	19.96308	19.94862	19.97094	19.82953	19.99724	19.98593	20.51843

[a]*miRTC: miRNA reverse transcription control miScript Primer Assay*
[b]*PPC: Positive PCR control*

Supplementary Table S10: Longitudinal Real-time RT-PCR cycle threshold (Ct) values of 84 cardio-miRs in responder patients

miRNA ID	Responders 6 Months													
	1	2	3	4	5	6	7	8	9	10	11	12	13	14
hsa-let-7a-5p	19.93017	19.93011	22.78725	20.32926	23.09488	23.97471	20.13568	24.93199	18.18389	23.98955	23.94663	22.4442	24.12062	25.31159
hsa-let-7b-5p	19.83429	19.95703	20.11079	19.90862	21.95568	21.64418	18.65608	23.44397	17.49196	21.65863	22.49703	19.32737	22.16759	23.95952
hsa-let-7c-5p	19.60794	22.29036	24.54212	23.80125	25.35441	26.10497	22.28011	27.46947	20.71601	25.79901	26.77693	23.99708	26.09353	27.55942
hsa-let-7d-5p	18.95534	22.36619	22.3888	20.47298	22.91474	24.44578	19.69541	24.65251	19.01866	23.71139	23.91538	21.92411	25.31337	25.48794
hsa-let-7e-5p	31.21359	-	23.98442	21.84344	24.31791	25.29718	20.6571	26.34134	19.28378	25.3658	24.97085	23.6471	24.98702	25.97999
hsa-let-7f-5p	30.88454	32.26915	25.45784	22.58489	25.17632	26.77902	21.5689	27.17022	19.53549	26.66564	25.93347	25.88668	26.4605	27.97553
hsa-miR-1-3p	28.14775	30.36403	29.82095	27.70556	30.16509	31.83696	24.78352	31.78098	24.33529	30.49525	29.90890	30.83147	32.76384	30.1259
hsa-miR-100-5p	-	-	25.67687	23.97522	25.80443	26.96623	23.05364	27.79986	23.13535	26.61417	26.96769	25.89462	28.53353	28.30471
hsa-miR-103a-3p	29.21449	32.94072	27.62163	25.66474	26.12881	29.09419	23.43541	29.07121	23.88089	28.32657	29.77333	27.12953	30.3893	29.71575
hsa-miR-107	29.91138	33.73687	31.74035	29.54726	30.33554	31.87339	26.4868	32.82658	26.10708	31.7825	30.97189	30.7459	32.8028	32.24168
hsa-miR-10b-5p	-	39.01448	28.12613	26.96309	30.00126	30.55687	26.99089	29.56714	26.00351	29.21344	31.65527	29.39976	30.57792	31.11409
hsa-miR-122-5p	35.163	-	21.10832	23.18134	26.46167	23.5958	20.06155	26.55157	21.63254	22.08557	24.67635	23.43155	24.99981	26.2295
hsa-miR-124-3p	25.86868	31.0516	28.98376	29.34979	29.55179	29.06952	29.61445	30.19957	27.79464	30.22184	29.23860	30.1013	29.74442	29.49319
hsa-miR-125a-5p	29.54444	33.89471	23.9888	22.23159	23.8978	26.09452	20.46884	26.12578	21.37054	25.95624	25.68563	23.70782	26.45071	26.91657
hsa-miR-125b-5p	24.10437	34.61625	26.59994	26.44754	26.38152	28.49526	23.96954	29.25108	24.0705	27.03002	28.98349	25.66426	29.48713	29.96062
hsa-miR-126-3p	17.36413	23.79737	22.0737	20.55354	21.3618	23.74145	18.3483	23.09809	18.27686	22.5673	22.50283	21.55562	24.87621	24.80891
hsa-miR-130a-3p	25.72138	31.85089	26.51933	26.33721	26.32544	27.90115	23.40592	27.51737	23.10896	26.72094	28.10915	25.17505	28.64093	30.18002
hsa-miR-133a-3p	30.39383	35.56599	33.96691	35.94566	33.95176	-	36.57546	-	33.07064	35.19397	35.31496	35.17661	-	35.53863
hsa-miR-133b	31.95796	33.74579	35.63522	35.98869	36.68855	-	35.19523	-	32.6754	-	-	-	35.69682	34.90822
hsa-miR-140-5p	15.07702	19.90829	30.12058	29.22608	29.06691	31.40444	26.33226	31.32969	25.29692	29.82215	30.55678	28.98363	33.50126	32.9315
hsa-miR-142-3p	17.71025	27.01809	27.77753	26.29296	26.16684	29.24478	22.9935	27.13976	22.35668	26.91284	27.40596	27.93166	29.92944	31.45441
hsa-miR-143-3p	23.42953	32.62117	31.15984	30.25065	28.02654	31.41131	26.25158	32.6585	26.11366	30.68804	31.25457	28.77698	32.33784	32.72685
hsa-miR-144-3p	27.13186	33.48467	28.98316	28.81325	27.93071	29.32117	25.0166	27.98801	22.86458	26.56941	29.64715	26.22014	30.79912	31.40867

hsa-miR-145-5p	27.07405	-	28.20038	27.03226	27.20499	27.95068	24.93947	28.32221	25.27052	27.26218	28.01861	27.40745	28.96265	30.06976
hsa-miR-146a-5p	27.20551	32.15971	24.9942	21.44378	23.24762	25.98969	20.06906	24.9109	20.15443	25.90174	23.50712	24.73187	27.09098	26.85355
hsa-miR-149-5p	23.44634	28.86465	37.73655	-	-	-	-	-	-	-	39.67019	-	-	-
hsa-miR-150-5p	26.07663	27.07568	19.94347	19.99118	23.37594	21.87374	20.11203	24.03684	19.19771	21.91632	22.96607	21.05597	21.39139	23.64383
hsa-miR-155-5p	20.92066	25.89037	29.2374	27.091	30.81157	30.70224	26.73587	33.36755	26.13795	31.764	30.08973	30.23782	31.92035	31.47224
hsa-miR-15b-5p	30.6529	33.70945	22.14334	19.93451	21.7685	23.46861	18.6668	23.4742	18.16314	22.59919	22.48551	21.48839	23.61055	24.71685
hsa-miR-16-5p	22.96395	30.90082	19.81707	20.2914	19.65659	20.79956	16.69479	21.17629	15.32465	19.2446	21.43558	17.963	21.28449	23.59954
hsa-miR-106a-5p hsa-miR-17-5p	23.37627	30.72098	26.26453	25.55759	24.78093	26.84026	22.36312	27.83134	21.3133	25.60956	27.27818	24.59082	28.18799	29.48805
hsa-miR-181a-5p	25.35605	32.31907	31.16294	29.22144	29.93254	30.42588	28.32558	32.65822	26.89761	30.86896	31.20465	30.46519	34.54178	34.86083
hsa-miR-181b-5p	22.83611	31.35027	26.67393	25.7715	27.71618	28.53551	24.989	28.81018	23.52884	28.1671	28.72375	26.70495	29.72535	29.56185
hsa-miR-182-5p	-	-	29.04596	27.8347	31.24586	32.53423	27.29136	32.37645	26.54788	30.75239	30.26219	30.97834	29.80687	33.11051
hsa-miR-183-5p	-	36.19307	27.26519	28.72326	29.58946	29.68921	26.3862	33.12299	24.20945	28.43538	30.02598	27.84569	29.12449	30.9404
hsa-miR-185-5p	25.17785	32.40368	25.69059	25.30199	25.73549	27.13495	21.77237	27.97886	20.76992	25.4489	27.70761	24.25588	28.33138	29.62051
hsa-miR-18b-5p	26.98813	34.48228	30.85575	27.23007	29.8755	29.46506	26.78431	31.04467	26.0542	30.72802	29.08144	29.7288	30.5004	30.84355
hsa-miR-195-5p	25.43114	31.22993	20.49792	20.96025	20.31915	21.36588	17.28935	21.92772	16.17171	20.06555	22.10033	18.68513	22.66393	24.40635
hsa-miR-199a-5p	24.10951	30.42263	-	29.39029	29.50529	33.38191	26.16558	30.45889	26.63561	30.99799	31.49853	31.25109	-	35.28498
hsa-miR-206	22.04993	31.64445	28.40022	30.08054	30.4422	30.40902	29.50797	29.78148	28.49776	30.59755	31.31379	28.35464	31.56122	32.92329
hsa-miR-208a-3p	24.16023	28.08858	34.47503	-	-	-	-	-	-	35.33765	-	-	-	-
hsa-miR-208b-3p	22.51136	26.54548	36.2518	-	34.78608	-	38.89011	-	-	-	-	-	-	-
hsa-miR-21-5p	19.80391	26.02469	20.13024	18.22924	20.11253	21.51456	17.17592	21.40077	16.14753	21.40945	21.18372	19.9619	21.85162	22.44066
hsa-miR-210-3p	24.16873	31.61115	29.64614	30.65934	32.90135	30.50999	29.52586	31.11912	28.95047	30.83035	31.42546	30.42785	30.24523	31.01924
hsa-miR-214-3p	23.71458	29.30463	30.99845	32.32989	35.58395	35.28051	29.97543	32.05806	30.07356	-	32.20391	31.84088	35.67217	33.97028
hsa-miR-22-3p	20.93823	25.9435	25.37802	25.35955	24.9265	26.66652	22.25277	26.90019	21.54239	25.73899	27.00589	23.88407	29.02664	29.57605
hsa-miR-221-3p	30.51735	31.30745	26.4949	23.12239	23.35901	27.47437	21.31717	25.6122	21.36353	26.60855	24.99229	24.97558	28.94979	28.18423
hsa-miR-222-3p	20.32279	25.5316	24.71054	22.75731	23.98357	26.49528	21.3113	25.82744	20.93269	25.75152	25.16836	24.2477	27.80387	27.9106
hsa-miR-223-3p	23.69862	30.99315	19.8501	17.00426	18.7611	21.20075	15.1716	21.17172	14.98715	20.29121	19.38201	19.49421	20.36603	21.298
hsa-miR-224-5p	24.54251	31.25227	30.16747	25.0166	25.93158	31.99454	25.10411	27.82652	22.78419	30.08027	28.43651	28.85817	31.38014	29.62449

hsa-miR-23a-3p	22.97819	32.46922	21.50987	18.47846	20.67834	22.89522	17.31827	22.33844	17.37582	22.1853	21.38811	21.19147	23.30134	23.01502
hsa-miR-23b-3p	30.53204	35.5716	23.95887	21.38124	23.8474	25.95876	20.76001	25.41549	20.68184	25.20515	24.66219	24.26645	26.71513	26.56813
hsa-miR-24-3p	30.41859	31.95936	24.72851	22.55684	23.01198	26.06339	20.10285	25.23285	20.42396	25.07601	24.95272	24.15222	27.79774	27.0654
hsa-miR-25-3p	18.57783	23.61178	20.30212	19.9305	21.16199	21.6005	17.88064	22.76178	16.57944	21.17814	22.23244	19.24795	21.95193	23.71634
hsa-miR-26a-5p	36.32061	36.1099	21.37821	18.94721	21.08862	23.98923	17.93744	23.34215	17.29604	22.90632	22.15649	22.31592	24.44178	24.25825
hsa-miR-26b-5p	35.98682	35.93759	22.81866	20.29673	22.77663	24.87558	19.38868	25.20206	17.97347	23.78514	23.83220	23.51105	25.0365	25.88174
hsa-miR-27a-3p	31.06448	29.64097	23.74556	22.97613	23.35426	25.01331	20.24414	24.69966	20.08678	24.02482	24.57244	22.94837	26.75629	26.81873
hsa-miR-27b-3p	30.16264	-	24.94802	23.8335	24.36565	26.72427	21.26946	26.18274	21.20182	25.33819	25.49470	24.86639	27.31816	27.73366
hsa-miR-29a-3p	20.37791	30.68022	25.94033	25.88494	26.74145	26.74643	23.17089	27.50304	22.77501	25.59758	27.27762	25.5125	27.82874	29.25669
hsa-miR-29b-3p	29.96895	29.933	25.01404	25.68853	28.05454	27.3012	23.70924	29.01705	22.22741	25.88455	28.07734	24.95304	27.52182	30.24283
hsa-miR-29c-3p	24.14598	32.51637	24.84299	24.95012	25.87211	26.23411	22.37152	27.45581	21.19302	24.9386	26.73358	24.11314	27.24019	29.38214
hsa-miR-302a-3p	29.47895	35.00066	36.19143	-	-	36.69648	-	-	-	-	-	-	36.01073	-
hsa-miR-302b-3p	30.34352	31.81234	-	-	-	-	-	-	33.82908	34.84256	-	-	35.90228	-
hsa-miR-30a-5p	25.24681	30.34427	24.42332	22.66556	23.02277	25.35802	20.11867	25.14254	19.98415	24.68618	24.76697	23.30667	26.55613	27.48351
hsa-miR-30c-5p	29.79206	-	25.24623	22.32018	24.17008	26.81529	21.33205	25.91426	20.62021	25.522	25.09430	25.62876	27.35299	27.77192
hsa-miR-30d-5p	24.92859	32.88781	25.04517	22.88268	23.47025	25.93751	20.51163	25.68036	20.69963	25.69295	25.16578	23.78949	26.92672	27.66972
hsa-miR-30e-5p	19.66148	30.13499	24.67107	23.18449	23.56135	25.89635	20.63051	25.38102	20.34683	24.81851	25.25399	23.58528	27.05286	27.95974
hsa-miR-31-5p	22.62977	26.92727	32.27045	33.37035	32.84113	33.02276	31.76892	35.11632	30.6517	32.2333	31.76875	34.65825	31.08982	32.34542
hsa-miR-320a	30.59409	31.78416	23.22719	21.05635	22.66095	24.55629	20.15779	24.45537	19.26395	24.84064	24.21487	22.40553	25.07976	25.37974
hsa-miR-328-3p	21.83035	25.06771	25.92071	25.93367	26.6424	26.96506	24.85606	27.95913	24.57533	26.99701	27.01760	28.20644	25.69667	27.13356
hsa-miR-342-3p	34.0864	-	22.79533	22.21283	25.08369	24.29408	21.78333	25.85519	21.07618	24.31419	25.00080	23.54281	24.59268	26.51143
hsa-miR-365a-3p hsa-miR-365b-3p	24.49236	30.66973	26.96491	26.9951	27.71695	28.83181	24.91408	30.93908	25.48456	27.78619	29.86240	27.81836	30.38093	31.74984
hsa-miR-378a-3p	28.2989	29.91406	29.44195	28.80047	28.90866	30.54787	25.64574	29.76385	25.60386	29.58466	31.20947	28.42261	31.37001	34.67785
hsa-miR-423-3p	25.64837	33.05162	28.91122	25.75956	25.75579	29.01913	24.06407	28.66069	23.95883	29.41148	27.90967	28.31027	30.26495	30.3758
hsa-miR-424-5p	30.34589	-	25.16054	24.78397	25.47731	26.2241	21.946	25.73388	20.74836	25.26629	25.83964	23.72261	27.16743	28.68435
hsa-miR-451a	26.99316	34.58776	18.03493	19.00587	18.41604	18.94462	14.94953	19.40052	13.64087	17.02367	20.17976	15.82266	20.3753	22.43438
hsa-miR-486-5p	31.40771	-	17.02238	16.53579	18.15936	18.42992	15.8651	19.56868	14.07065	18.21765	19.49836	16.62521	18.4887	20.13332

hsa-miR-494-3p	33.33674	-	32.58527	28.08669	28.28501	32.34299	25.76612	33.93539	27.44402	30.96150	30.36043	31.00075	35.09527	33.96811
hsa-miR-499a-5p	34.69484	-	31.90957	33.55568	34.22645	-	30.27990	34.96983	30.34163	32.74298	35.69646	34.36368	-	36.9164
hsa-miR-7-5p	25.79160	36.10832	25.38497	26.57358	26.65127	27.12324	23.50724	28.29801	22.20227	26.71575	28.38017	25.16880	27.35941	29.80071
hsa-miR-92a-3p	22.10689	29.10377	18.93948	18.08225	19.38331	20.13443	16.99602	20.95013	15.87147	20.22044	20.95834	18.12459	20.67945	22.06932
hsa-miR-93-5p	24.77215	34.51086	25.10185	24.89512	24.01542	25.90282	21.41111	26.80659	20.88216	24.50971	25.88717	23.58176	27.92697	28.81531
hsa-miR-98-5p	24.42135	30.57770	28.95216	25.17151	27.13505	-	23.98332	29.21983	23.73051	30.20281	28.21214	29.23187	30.98427	29.87256
hsa-miR-99a-5p	29.03651	30.01373	26.64364	24.98177	26.95329	28.15083	24.27035	28.49957	24.32134	27.73484	28.34909	26.95835	29.45461	29.5557
SNORD61	29.60881	-	28.4649	31.00081	32.98455	30.91636	32.00243	32.32595	30.53066	31.90538	33.82918	31.56787	33.6024	32.57404
SNORD68	26.97070	34.58306	25.27023	30.99777	30.55224	29.10997	29.05469	-	28.50172	29.13354	33.36228	29.75993	28.51457	32.40304
SNORD72	24.73398	30.40427	36.82988	34.96505	-	-	-	-	35.33326	-	-	-	-	-
SNORD95	29.42813	31.04096	25.74819	30.36941	29.97839	26.96482	26.90249	31.95966	27.10395	28.44204	31.63596	27.16495	26.96741	32.78776
SNORD96A	24.60286	28.22510	27.62502	30.37678	30.23673	29.13903	28.53485	-	27.67612	29.86049	30.60321	27.80721	28.96089	32.29509
RNU6-6P	23.65303	26.84755	28.91076	31.91758	31.27625	33.11799	30.13059	33.67167	29.43722	31.61308	32.41977	31.10894	30.99949	-
miRTC[a]	23.05959	26.83156	19.24074	19.80461	19.03664	19.04556	19.18776	19.11975	18.5493	18.9115	19.26188	19.59476	21.62903	21.95611
miRTC[a]	25.00643	28.04658	19.27727	19.67604	19.10458	18.96431	19.20060	19.05637	18.59531	18.76497	19.15024	19.49967	21.81584	22.17700
PPC[b]	20.41120	23.37674	19.70755	20.05218	19.68398	20.05203	19.98837	19.85644	20.03064	19.90418	19.92544	19.92142	20.01525	20.61062
PPC[b]	22.96076	25.95099	19.76407	19.98144	20.05076	20.21896	19.88278	20.02878	19.99028	19.8882	20.07138	19.86375	20.08341	20.60325

[a]*miRTC: miRNA reverse transcription control miScript Primer Assay*
[b]*PPC: Positive PCR control*

Supplementary Table S11: Basal Real-time RT-PCR cycle threshold (Ct) values of 84 cardio-miRs in non-responder patients

miRNA ID	Non-responders Baseline										
	1	2	3	4	5	6	7	8	9	10	11
hsa-let-7a-5p	24.81133	26.30694	25.65788	24.4667	22.14805	24.71253	24.2067	25.38032	16.4496	27.64415	27.38487
hsa-let-7b-5p	23.98296	25.28884	23.91495	22.88947	20.22782	21.17705	23.40913	23.6377	15.26021	24.17576	26.34651
hsa-let-7c-5p	27.15254	29.19334	27.68124	26.30528	23.50439	26.47447	27.33071	27.52283	18.42329	28.82802	28.38993
hsa-let-7d-5p	25.91651	27.04972	25.83116	24.79937	22.20951	25.90759	24.99597	26.82879	16.31152	31.48145	28.92346
hsa-let-7e-5p	25.94347	27.94615	26.71169	25.63599	23.03729	25.49018	25.0419	26.3096	17.27608	28.16909	27.82645
hsa-let-7f-5p	26.97403	28.61715	27.97735	26.80365	24.87944	28.4242	25.59598	28.36728	18.33251	31.43927	29.99677
hsa-miR-1-3p	32.23328	32.25077	-	28.60281	25.50664	34.44957	31.72326	34.0499	25.39873	-	33.19689
hsa-miR-100-5p	26.91872	28.14354	28.37645	25.65108	23.67261	28.9379	26.82527	29.53639	23.39071	32.87994	30.60648
hsa-miR-103a-3p	30.24552	32.69909	31.24978	30.13565	28.26304	31.94693	28.97943	32.24696	20.74489	35.48483	33.58417
hsa-miR-107	32.01397	35.57991	33.48289	32.28994	31.53936	-	33.97409	-	22.61087	33.29326	-
hsa-miR-10b-5p	29.70444	30.96512	31.29209	31.88744	26.71885	32.07006	29.89514	30.95919	28.77866	-	-
hsa-miR-122-5p	26.51262	26.72275	26.90499	22.9863	18.39364	25.32844	25.67178	27.12698	19.9373	29.59958	27.66712
hsa-miR-124-3p	28.40925	28.30576	29.43064	29.47168	30.38575	31.05756	29.36676	29.71089	29.34833	29.31572	29.59002
hsa-miR-125a-5p	26.1123	27.7088	27.03022	25.21437	22.68647	27.21111	26.1851	27.54705	21.51426	32.29156	28.79344
hsa-miR-125b-5p	28.00442	29.0704	28.77772	26.40038	23.63028	30.59306	28.12024	30.99709	22.82449	-	31.67853
hsa-miR-126-3p	24.78526	25.15352	24.96992	22.86982	21.19662	26.95844	24.31072	26.48452	17.8553	30.94523	29.04055
hsa-miR-130a-3p	29.52003	30.18685	29.92437	28.53514	26.54833	31.5914	29.28011	32.38491	20.61889	-	-
hsa-miR-133a-3p	32.49273	35.97459	37.10251	34.93331	34.46545	-	39.78593	35.7001	32.67762	-	-
hsa-miR-133b	32.44858	-	-	-	35.66105	-	-	-	31.55307	-	34.90721
hsa-miR-140-5p	32.96822	32.22116	35.21446	31.19728	29.60947	-	31.96344	-	25.03494	-	-
hsa-miR-142-3p	30.49862	29.97235	30.19386	28.20863	25.67191	35.08347	28.53223	36.63636	22.94332	33.24833	34.73325
hsa-miR-143-3p	33.71299	-	30.68604	29.68711	28.21034	-	-	-	28.94549	-	-

hsa-miR-144-3p	29.86632	30.62327	30.03228	29.44157	26.48570	-	32.18331	32.54567	21.15223	-	33.16713
hsa-miR-145-5p	27.52685	27.9099	27.82154	28.25653	27.28841	30.4266	28.12468	31.04837	26.09296	34.89249	31.89951
hsa-miR-146a-5p	28.0098	28.62566	26.97116	25.19818	24.05903	29.31953	25.42715	30.63269	20.94795	-	32.40827
hsa-miR-149-5p	33.44521	-	-	-	36.60941	-	-	-	34.71621	-	-
hsa-miR-150-5p	24.10813	25.05183	24.93781	24.91524	20.05791	24.4205	22.71652	25.87205	21.52781	29.50717	26.70975
hsa-miR-155-5p	30.30973	32.21333	32.24989	32.66084	28.94461	29.54	31.73182	35.42041	25.96631	31.12611	33.10757
hsa-miR-15b-5p	26.33285	26.79107	24.139	23.37335	21.64610	26.26671	23.70287	25.51365	15.92379	30.22613	27.89351
hsa-miR-16-5p	25.1401	24.78912	22.99171	21.34988	19.30497	26.24649	23.91134	23.39925	13.82831	30.6075	27.07226
hsa-miR-106a-5p hsa-miR-17-5p	28.85089	29.62017	29.11628	27.63202	24.90981	32.53523	28.19453	30.04052	19.81447	32.73845	29.86911
hsa-miR-181a-5p	32.41935	34.11472	31.50655	32.63215	30.31976	-	30.15489	33.74017	24.83147	-	-
hsa-miR-181b-5p	28.71326	30.12893	29.89444	28.60162	26.00384	29.04827	28.06042	31.0819	22.92291	32.77547	35.60949
hsa-miR-182-5p	29.73073	31.34663	30.23283	31.20394	29.25071	33.34258	28.95607	34.60933	21.08743	-	-
hsa-miR-183-5p	31.51597	33.7606	33.83264	32.55724	28.32008	31.43906	30.36949	31.12971	19.22233	33.92004	31.68056
hsa-miR-185-5p	29.92359	30.00428	28.30173	27.96551	24.49253	31.14012	28.21471	30.03633	18.57515	32.49574	34.47882
hsa-miR-18b-5p	30.29969	33.60613	29.90021	30.01932	30.41582	32.33982	28.88838	32.45915	24.72671	32.95627	33.24069
hsa-miR-195-5p	25.68329	25.4401	23.62489	21.91696	20.19385	26.86106	21.03572	23.96433	14.29516	32.10435	28.15205
hsa-miR-199a-5p	32.40623	33.45205	36.36146	-	31.90337	-	34.61956	-	26.76774	-	-
hsa-miR-206	29.71091	33.96214	31.11393	28.98229	29.33521	31.13818	32.94838	33.88058	32.12765	31.39993	31.93801
hsa-miR-208a-3p	33.08848	39.84809	-	-	-	-	-	38.15754	34.60326	-	-
hsa-miR-208b-3p	34.23346	34.72229	33.17939	-	32.20971	-	-	-	-	-	-
hsa-miR-21-5p	22.28981	23.99202	22.47824	20.6675	18.66760	22.66513	21.02494	23.36809	14.36016	27.85472	25.13013
hsa-miR-210-3p	30.63127	31.13582	32.13097	30.82009	30.21895	33.04581	33.40361	30.73591	26.95809	30.97937	31.34713
hsa-miR-214-3p	32.14843	32.61144	32.71647	33.24553	29.47375	-	33.41637	-	32.89993	34.75017	34.56253
hsa-miR-22-3p	27.95975	28.20288	27.61314	26.41494	24.71620	33.55685	27.7875	30.75035	20.57229	-	36.96518
hsa-miR-221-3p	28.47961	28.38276	28.14864	25.82673	25.54527	31.60608	26.65685	31.79063	21.24464	34.36667	31.64512
hsa-miR-222-3p	27.43324	28.39985	27.48103	25.86304	24.34161	30.0813	26.03216	29.02768	21.51697	32.30276	34.10348
hsa-miR-223-3p	22.32587	24.49268	21.9121	21.08635	19.16157	23.9543	21.83372	24.20052	15.21376	27.62321	25.38966

hsa-miR-224-5p	33.82536	31.02155	32.69709	30.38456	28.79813	30.94624	31.29113	34.49621	27.21017	-	33.03044
hsa-miR-23a-3p	23.96070	25.36056	23.55368	22.0212	20.43876	24.57974	22.73986	25.65508	17.40952	28.49796	26.39349
hsa-miR-23b-3p	26.12724	27.85608	26.92706	25.30584	22.98634	28.01727	25.69257	28.20532	21.54441	32.78288	29.98447
hsa-miR-24-3p	27.87268	28.25569	27.58412	24.96817	23.61540	32.38477	26.8217	30.09324	20.7131	-	31.93586
hsa-miR-25-3p	24.33797	25.27452	22.71884	22.59748	19.64668	23.27559	22.53571	23.21772	15.38526	30.98148	25.56112
hsa-miR-26a-5p	24.47412	25.9563	25.63191	24.08385	21.74973	27.70859	23.16214	27.0957	16.42122	30.99329	27.68921
hsa-miR-26b-5p	25.15673	27.32159	26.93628	25.36018	22.82048	27.65416	24.28128	27.20072	16.71431	32.22248	28.55926
hsa-miR-27a-3p	25.97878	26.95006	25.93308	24.2232	21.95552	29.71416	25.50834	28.35891	19.67862	-	30.5935
hsa-miR-27b-3p	27.66308	27.84571	27.66365	25.41401	22.34634	29.98115	26.64659	29.46879	21.08978	-	33.15234
hsa-miR-29a-3p	28.19929	28.85526	28.88099	27.03975	24.39931	29.84066	28.56586	30.10189	22.32036	31.60816	29.82602
hsa-miR-29b-3p	30.88939	30.5407	30.97452	28.34858	25.63511	31.7478	28.62694	29.64136	19.55225	-	31.34174
hsa-miR-29c-3p	28.91687	29.08324	28.46128	26.67847	23.79477	31.91851	28.00257	29.44222	19.54223	-	31.93087
hsa-miR-302a-3p	33.87630	-	-	32.20512	33.62856	-	-	-	-	-	37.02446
hsa-miR-302b-3p	-	-	-	36.46421	36.59692	-	-	-	31.39902	-	-
hsa-miR-30a-5p	26.60989	27.91821	26.94485	25.1229	22.93262	30.23616	25.45955	28.28597	19.32807	31.78558	31.44625
hsa-miR-30c-5p	26.65981	27.81996	28.20612	26.6077	24.91205	30.17896	26.2557	30.11908	21.90987	32.90091	32.17997
hsa-miR-30d-5p	27.23291	28.35089	27.80882	25.9215	23.55762	30.17543	26.00007	28.72974	19.81652	31.63093	31.77824
hsa-miR-30e-5p	26.55431	27.96208	27.01433	25.48366	23.19162	30.07314	25.95978	28.97635	19.56278	31.72754	31.34951
hsa-miR-31-5p	31.06966	32.32522	31.54013	34.30622	31.63590	32.34285	34.37337	31.50013	31.34266	31.50536	32.41733
hsa-miR-320a	25.42797	26.93314	25.30085	24.74474	21.64691	24.09352	23.96898	26.30102	16.93461	28.0282	30.24259
hsa-miR-328-3p	24.59361	26.49342	27.06734	27.15777	26.82253	27.85247	26.74234	26.84609	25.61341	27.58392	27.09417
hsa-miR-342-3p	25.94322	27.1769	26.85812	26.45754	22.67694	28.24647	25.5094	27.89616	22.18208	28.66249	29.06575
hsa-miR-365a-3p hsa-miR-365b-3p	30.52937	32.09037	30.51400	28.41197	24.29078	31.44261	30.40864	31.09541	26.07396	-	33.09603
hsa-miR-378a-3p	30.61658	31.41964	34.68985	31.8586	27.44928	32.87674	32.59548	33.23118	23.68012	-	-
hsa-miR-423-3p	29.67415	29.96766	29.96476	30.18772	28.26161	32.43334	29.02464	31.17397	25.20677	32.43642	32.43654
hsa-miR-424-5p	27.92038	28.23139	27.54185	25.88857	23.52522	31.97377	27.58109	30.54012	19.94684	33.09068	31.9271
hsa-miR-451a	23.41384	22.48264	21.96687	19.72102	17.22226	24.71068	21.69097	22.47973	12.37459	25.39588	26.64019

hsa-miR-486-5p	18.6216	19.93651	18.95077	19.51172	16.71562	18.72106	18.4695	19.23424	12.33385	25.28271	21.52177
hsa-miR-494-3p	29.9265	32.52657	36.15216	30.25745	30.00187	32.88005	30.73361	35.02886	29.2981	34.34922	34.45171
hsa-miR-499a-5p	33.72573	33.95618	33.93571	33.50202	29.10333	-	-	-	30.79617	-	-
hsa-miR-7-5p	30.24173	32.22461	29.10503	28.97482	25.27816	28.53309	27.99922	28.33868	19.30747	35.50727	31.11555
hsa-miR-92a-3p	21.16287	22.71352	21.27324	21.29853	18.45877	22.92415	20.50925	21.95515	14.83917	29.41874	24.9344
hsa-miR-93-5p	29.524	28.82168	28.03325	26.84008	24.22874	31.1155	27.71897	29.45488	18.99104	-	35.74141
hsa-miR-98-5p	29.78519	32.88033	31.32006	29.66981	28.35761	32.58984	29.71962	37.41221	23.95353	34.54317	-
hsa-miR-99a-5p	27.94616	29.11279	29.31061	26.81895	24.84456	29.78689	27.86173	30.30285	24.68749	33.7638	31.13881
SNORD61	29.05884	32.051	32.66017	32.00511	29.37387	35.36753	31.4549	34.25304	29.85088	-	36.94406
SNORD68	33.83559	34.81919	33.27018	-	27.80394	33.50906	30.6112	-	28.12391	-	-
SNORD72	35.27882	-	-	-	-	-	-	36.48182	36.6035	-	-
SNORD95	30.80504	33.25209	33.30819	30.53891	27.81444	-	30.33563	32.92751	26.93055	-	-
SNORD96A	31.78437	35.3471	33.17014	32.04503	29.29656	-	31.25276	-	28.18251	34.69247	35.00496
RNU6-6P	31.10193	32.15644	-	32.49541	30.62344	-	33.73945	34.90786	31.08695	-	-
miRTC[a]	18.78634	19.08893	19.29626	19.1168	19.35296	21.9569	19.69357	22.02296	21.66853	27.27509	24.27622
miRTC[a]	18.8164	18.66931	19.19238	19.31367	19.28120	22.02626	19.53645	21.8936	21.81053	27.31419	24.10427
PPC[b]	19.86878	19.94911	19.76684	20.12112	19.69929	19.94468	19.96608	19.93345	20.01198	20.03554	20.36809
PPC[b]	19.72506	20.0478	19.70191	19.82953	19.97094	19.94942	20.05788	19.90446	20.04762	20.09036	20.53596

[a]*miRTC: miRNA reverse transcription control miScript Primer Assay*
[b]*PPC: Positive PCR control*
[c]*patient died before 6 -month revision*

Supplementary Table S12: Longitudinal Real-time RT-PCR cycle threshold (Ct) values of 84 cardio-miRs in non-responder patients

miRNA ID	Non-responders 6 Months										
	1	2	3	4	5	6	7[c]	8[c]	9	10	11
hsa-let-7a-5p	20.67119	18.10254	23.75598	23.94663	23.98955	24.24961	-	-	21.42821	25.93251	24.26432
hsa-let-7b-5p	19.38917	15.90552	22.15835	22.49703	21.65863	22.29983	-	-	20.54238	24.54473	21.98201
hsa-let-7c-5p	23.08038	20.21245	26.16904	26.77693	25.79901	26.45609	-	-	23.84579	28.09997	25.97902
hsa-let-7d-5p	21.99577	18.05562	23.67769	23.91538	23.71139	23.20974	-	-	22.21428	26.79273	24.64337
hsa-let-7e-5p	21.86782	19.73669	24.86004	24.97085	25.36580	25.18003	-	-	22.25653	26.85381	25.11693
hsa-let-7f-5p	22.59794	21.84737	25.92720	25.93347	26.66564	26.40219	-	-	23.82061	28.4781	26.94483
hsa-miR-1-3p	28.15801	27.80458	30.28876	29.9089	30.49525	31.28629	-	-	29.43527	32.8245	32.57249
hsa-miR-100-5p	24.81277	23.63691	26.24435	26.96769	26.61417	27.04036	-	-	25.78295	31.77821	27.56201
hsa-miR-103a-3p	25.80979	24.3809	28.32154	29.77333	28.32657	27.7055	-	-	28.11699	31.20572	31.02114
hsa-miR-107	27.28321	26.66047	30.35052	30.97189	31.78250	30.71893	-	-	30.84776	32.58277	34.08821
hsa-miR-10b-5p	26.72349	26.97889	29.18225	31.65527	29.21344	29.05681	-	-	28.35636	31.73658	30.23461
hsa-miR-122-5p	21.80654	22.34752	23.73457	24.67635	22.08557	24.5108	-	-	21.96225	26.30843	24.6585
hsa-miR-124-3p	27.17551	29.49303	29.65023	29.2386	30.22184	29.38802	-	-	29.61673	28.9314	29.69263
hsa-miR-125a-5p	23.10409	22.86601	25.17866	25.68563	25.95624	24.53546	-	-	23.54371	28.10593	25.98127
hsa-miR-125b-5p	25.81450	24.3023	26.9099	28.98349	27.03002	26.95588	-	-	26.9888	31.20733	28.98571
hsa-miR-126-3p	20.96709	19.46654	21.93995	22.50283	22.56730	22.38423	-	-	22.41202	26.95906	24.7959
hsa-miR-130a-3p	25.12292	21.62851	26.64352	28.10915	26.72094	27.09042	-	-	27.74039	31.73499	29.01722
hsa-miR-133a-3p	33.61233	-	33.99025	35.31496	35.19397	37.07318	-	-	39.10155	-	38.50649
hsa-miR-133b	33.77158	38.79407	35.10856	-	-	-	-	-	34.16043	-	34.45572
hsa-miR-140-5p	28.37843	26.6752	30.37507	30.55678	29.82215	29.61173	-	-	30.33461	-	33.30502
hsa-miR-142-3p	26.38321	26.99388	28.48666	27.40596	26.91284	27.20276	-	-	27.46846	31.77939	30.87302
hsa-miR-143-3p	27.52182	29.1436	30.58659	31.25457	30.68804	31.24981	-	-	32.25723	-	32.75704

hsa-miR-144-3p	25.71508	23.92382	27.33897	29.64715	26.56941	28.97353	-	-	28.82550	30.81448	29.47142
hsa-miR-145-5p	26.68017	27.83815	27.56442	28.01861	27.26218	28.40628	-	-	29.18294	29.63219	30.34374
hsa-miR-146a-5p	23.58296	22.33726	24.14318	23.50712	25.90174	24.88151	-	-	24.91604	31.82187	28.13587
hsa-miR-149-5p	-	-	-	39.67019	-	-	-	-	-	-	33.6819
hsa-miR-150-5p	20.12927	19.44654	22.93977	22.96607	21.91632	21.37614	-	-	20.73296	25.97893	22.22126
hsa-miR-155-5p	27.75776	28.74687	30.11877	30.08973	31.76400	31.97867	-	-	28.53275	33.18516	32.44494
hsa-miR-15b-5p	21.34112	17.3111	22.19683	22.48551	22.59919	22.43958	-	-	22.14326	26.33858	24.41146
hsa-miR-16-5p	18.23173	15.10747	19.23685	21.43558	19.24460	20.05057	-	-	20.63400	24.1394	21.5239
hsa-miR-106a-5p hsa-miR-17-5p	24.04849	21.37066	25.96406	27.27818	25.60956	26.09411	-	-	27.38828	31.61787	28.06207
hsa-miR-181a-5p	28.75554	28.5161	31.6327	31.20465	30.86896	30.08088	-	-	32.47554	32.46032	35.04068
hsa-miR-181b-5p	25.86926	23.24813	28.20375	28.72375	28.16710	27.32217	-	-	27.30159	31.891	28.70656
hsa-miR-182-5p	29.27318	25.94592	29.66649	30.26219	30.75239	31.65779	-	-	27.83245	31.71438	30.71989
hsa-miR-183-5p	26.99822	22.97217	29.45778	30.02598	28.43538	28.72161	-	-	26.48644	30.43638	28.04388
hsa-miR-185-5p	23.40529	20.2767	25.28329	27.70761	25.44890	26.53869	-	-	26.05976	30.7605	27.8626
hsa-miR-18b-5p	27.5803	27.30177	29.53677	29.08144	30.72802	30.16422	-	-	29.94535	35.36157	33.1069
hsa-miR-195-5p	18.95043	15.98473	19.96098	22.10033	20.06555	20.68194	-	-	21.25583	24.93004	22.5215
hsa-miR-199a-5p	30.61961	32.14219	30.03401	31.49853	30.99799	32.91945	-	-	31.94575	-	-
hsa-miR-206	30.74669	29.98842	30.53782	31.31379	30.59755	31.98948	-	-	31.29835	32.30148	31.49421
hsa-miR-208a-3p	35.81211	-	34.41933	-	35.33765	-	-	-	-	-	-
hsa-miR-208b-3p	35.81045	32.92782	36.36019	-	-	-	-	-	-	-	-
hsa-miR-21-5p	18.79363	16.04859	20.74878	21.18372	21.40945	20.68246	-	-	19.91817	23.96186	21.89272
hsa-miR-210-3p	29.49498	30.2469	31.09182	31.42546	30.83035	30.98382	-	-	30.1407	30.14078	30.52664
hsa-miR-214-3p	29.96683	29.71264	33.93565	32.20391	-	-	-	-	32.367	-	34.53548
hsa-miR-22-3p	24.07269	20.99017	24.39359	27.00589	25.73899	25.41677	-	-	27.07809	31.04381	28.39486
hsa-miR-221-3p	23.71901	22.40965	24.22673	24.99229	26.60855	25.47477	-	-	26.63779	33.21243	29.97151
hsa-miR-222-3p	22.95844	20.96665	24.97688	25.16836	25.75152	24.68369	-	-	25.44353	31.08191	27.50385
hsa-miR-223-3p	17.40850	17.95729	19.40686	19.38201	20.29121	19.29384	-	-	19.06306	24.45714	21.73299

hsa-miR-224-5p	27.28206	26.64782	28.49830	28.43651	30.08027	27.21929	-	-	30.49158	-	31.69626
hsa-miR-23a-3p	20.10557	19.28454	21.1107	21.38811	22.18530	21.35079	-	-	21.05217	26.1745	23.84112
hsa-miR-23b-3p	23.23867	21.82141	24.58714	24.66219	25.20515	24.13987	-	-	24.04287	29.72972	26.73794
hsa-miR-24-3p	23.36449	22.3228	23.96472	24.95272	25.07601	24.84631	-	-	24.94426	29.86362	27.65765
hsa-miR-25-3p	18.95297	15.06392	20.64244	22.23244	21.17814	20.53004	-	-	20.38542	24.16978	21.54683
hsa-miR-26a-5p	20.72313	19.74939	22.75047	22.15649	22.90632	21.86156	-	-	21.61098	27.24788	24.28947
hsa-miR-26b-5p	21.03555	20.18047	24.23211	23.83220	23.78514	23.15302	-	-	22.62446	27.90432	24.75421
hsa-miR-27a-3p	22.74397	21.07528	23.51771	24.57244	24.02482	24.39931	-	-	23.93456	28.86074	25.8675
hsa-miR-27b-3p	23.97501	22.54743	24.67872	25.49470	25.33819	25.98126	-	-	25.14972	29.76769	27.28793
hsa-miR-29a-3p	25.08566	24.69121	26.49275	27.27762	25.59758	26.46419	-	-	26.75188	29.0841	28.1708
hsa-miR-29b-3p	25.26571	22.86137	27.35209	28.07734	25.88455	25.99825	-	-	26.20848	30.37717	27.92126
hsa-miR-29c-3p	24.06257	22.64756	25.58276	26.73358	24.93860	25.40136	-	-	25.98595	29.58814	27.27649
hsa-miR-302a-3p	34.17590	-	34.31525	-	-	35.34549	-	-	37.30429	-	-
hsa-miR-302b-3p	35.34898	33.40471	34.17100	-	34.84256	-	-	-	34.71580	-	-
hsa-miR-30a-5p	22.60695	20.18355	23.85548	24.76697	24.68618	24.57494	-	-	25.16373	29.16998	26.62144
hsa-miR-30c-5p	23.17631	22.88563	25.19413	25.0943	25.52200	24.51542	-	-	25.25086	30.62834	27.97795
hsa-miR-30d-5p	22.98790	20.6515	24.44204	25.16578	25.69295	25.35846	-	-	25.35369	29.37332	26.99463
hsa-miR-30e-5p	22.87234	20.80281	23.91546	25.25399	24.81851	25.00339	-	-	25.63482	29.59474	27.08336
hsa-miR-31-5p	29.78541	31.74102	31.41723	31.76875	32.23330	32.24521	-	-	31.57814	31.44342	32.06218
hsa-miR-320a	21.94687	17.20836	23.72131	24.21487	24.84064	23.65791	-	-	22.62613	27.15543	24.18483
hsa-miR-328-3p	24.94798	26.00662	25.00233	27.01760	26.99701	27.26189	-	-	26.38899	26.98575	27.24166
hsa-miR-342-3p	22.98133	21.61814	24.7984	25.00080	24.31419	23.91124	-	-	24.09616	27.76762	25.49711
hsa-miR-365a-3p hsa-miR-365b-3p	26.65371	25.02785	28.99107	29.8624	27.78619	28.53593	-	-	27.22315	31.52575	30.33264
hsa-miR-378a-3p	27.17353	25.03761	29.09335	31.20947	29.58466	29.81866	-	-	29.94354	33.1348	32.5244
hsa-miR-423-3p	26.40878	25.01031	27.97436	27.90967	29.41148	28.58388	-	-	28.64653	31.69756	32.33381
hsa-miR-424-5p	24.81689	20.87552	24.77142	25.83964	25.26629	25.12033	-	-	25.53704	28.64404	26.95366
hsa-miR-451a	16.80462	13.21976	17.55974	20.17976	17.02367	17.82229	-	-	19.38987	22.77194	20.25308

hsa-miR-486-5p	15.96543	12.14002	17.99886	19.49836	18.21765	17.13469	-	-	17.17519	20.76943	17.64437
hsa-miR-494-3p	28.19548	30.3216	29.46409	30.36043	30.96150	30.14024	-	-	33.62355	33.97069	36.89389
hsa-miR-499a-5p	33.19468	30.90421	32.01321	35.69646	32.74298	37.64043	-	-	33.29376	37.8152	34.20096
hsa-miR-7-5p	24.85074	21.2627	26.98492	28.38017	26.71575	26.73566	-	-	26.96581	29.20069	27.03054
hsa-miR-92a-3p	17.57005	14.2926	19.68404	20.95834	20.22044	19.83546	-	-	19.30787	22.65953	20.16705
hsa-miR-93-5p	23.12982	19.94123	24.75322	25.88717	24.50971	25.11983	-	-	25.94066	30.11272	27.20244
hsa-miR-98-5p	27.80822	26.36992	28.8484	28.21214	30.20281	29.06903	-	-	27.22635	34.94385	32.14102
hsa-miR-99a-5p	25.8686	24.69474	27.16636	28.34909	27.73484	28.06504	-	-	26.69628	31.87579	28.92958
SNORD61	27.4108	28.83534	30.17895	33.82918	31.90538	30.87704	-	-	34.63133	35.63515	34.46188
SNORD68	25.61183	28.46518	32.39809	33.36228	29.13354	28.32876	-	-	32.92159	-	31.85387
SNORD72	36.12534	36.54549	34.9552	-	-	34.64566	-	-	-	-	-
SNORD95	24.95289	26.04479	32.00365	31.63596	28.44204	28.61174	-	-	30.12249	33.39253	31.17332
SNORD96A	28.07045	28.70503	30.94636	30.60321	29.86049	30.67915	-	-	29.90326	-	32.29078
RNU6-6P	26.51749	31.58734	31.15942	32.41977	31.61308	30.27643	-	-	32.93758	35.64695	35.01402
miRTC[a]	18.4449	20.01607	18.65918	19.26188	18.91150	19.57762	-	-	21.74536	21.40635	22.29729
miRTC[a]	18.42163	19.9491	18.57195	19.15024	18.76497	19.57963	-	-	21.94222	21.60959	22.17055
PPC[b]	19.83464	19.878	19.93641	19.92544	19.90418	20.10901	-	-	19.8953	20.02168	20.74307
PPC[b]	19.94724	19.87483	20.08621	20.07138	19.88820	19.7551	-	-	19.9417	20.08331	20.57839

[a] *miRTC: miRNA reverse transcription control miScript Primer Assay*
[b] *PPC: Positive PCR control*
[c] *patient died before 6 -month revision*
